# CaspNeuroD: a knowledgebase of predicted caspase cleavage sites in human proteins related to neurodegenerative diseases

**DOI:** 10.1093/database/baw142

**Published:** 2016-12-26

**Authors:** Sonu Kumar, Piotr Cieplak

**Affiliations:** SBP Medical Discovery Institute, 10901 North Torrey Pines Road, La Jolla, CA 92037, USA

## Abstract

**Background:** A variety of neurodegenerative diseases (NDs) have been associated with deregulated caspase activation that leads to neuronal death. Caspases appear to be involved in the molecular pathology of NDs by directly cleaving important proteins. For instance, several proteins involved in Alzheimer’s disease, including β-amyloid precursor protein (APP) and presenilins, are known to be cleaved by caspases. Therefore, cell death pathway may play a central role in many neurological diseases, and targeting the important proteins that control the cell survival and death may potentially represent a therapeutic approach for chronic neurodegenerative disorders.

**Findings:** We developed CaspNeuroD, a relational database of *in silico* predicted caspase cleavage sites in human proteins associated with NDs. The prediction has been done on collection of 249 human proteins reported in clinical studies of NDs using the recently published CaspDB Random Forest machine-learning model. This database could be used for identifying new caspase substrates and further our understanding of the caspase-mediated substrate cleavage in NDs.

**Conclusion:** Our database provides information about potential caspase cleavage sites in a verified set of human proteins involved in NDs. It provides also information about the conservation of cleavage positions in corresponding orthologs, and information about the positions of single nucleotide polymorphisms and posttranslational modifications (PTMs) that may modulate the caspase cleavage efficiency.

**Database URL:** caspdb.sanfordburnham.org/caspneurod.php .

## Introduction

Many neurodegenerative diseases (NDs), including brain trauma, Huntington's disease (HD), Parkinson’s disease, Alzheimer’s disease (AD), stroke, spinal cord injury and amyotrophic lateral sclerosis (ALS)—are associated with neuronal cell death (1). Necrosis and apoptosis are two main mechanisms of cell death (2–4). Necrotic cell death in the central nervous system follows acute ischemia or traumatic injury to the brain or spinal cord (5, 6). In contrast, apoptotic cell death, also known as programmed cell death, can be a feature of both acute and chronic neurologic diseases (1, 3, 7). In chronic NDs, it is the predominant form of cell death (8, 9). In apoptosis, a biochemical cascade activates proteases that destroy proteins, that are required for cell survival, and activates other types of proteins that mediate programmed cell death. Caspases actively contribute to the molecular pathogenesis of these diseases.

Caspases are proteolytic enzymes that perform hydrolysis of the peptide bonds in proteins to regulate their function in biological pathway(s), including the immune response, DNA replication, cell cycle progression, cell proliferation and apoptosis (10, 11). Until now, at least 15 distinct caspases have been identified in mammals (12). Human caspases are divided into apoptotic (Caspase-2, -3, -6, -7, -8, -9 and -10) and inflammatory (Caspase-1, -4 and -5) members. The apoptotic members have been further sub-divided into initiators (Caspase-2, -8, -9 and -10) and effectors (Caspase-3, -6 and -7) (13). The most prominent feature of caspase-specificity is that caspases cleave their substrates almost exclusively after Asp residues. The consensus cleavage motif, determined by analysis of known cleavage sites, is DXXD-G/A/S/T/N, pointing to the overlapping specificity of this family of enzymes (14–16).

During apoptosis, caspases initiate, coordinate and accelerate cell death and dismantling by cleaving crucial structural and enzymatic proteins. There are variety of ways in which caspase activity may contribute to chronic NDs such as HD and AD. One way is to eliminate damaged neurons that are beyond repair, which suggests, that cells can no longer cope with their toxic loads and caspase pathway is therefore activated. Importantly, several NDs are characterized by the accumulation of abnormal protein deposits, such as Aβ42 in senile plaques in AD and polyglutamine-containing aggregates in HD. An additional way by which caspase activity may contribute to neurodegeneration is generating toxic fragments from key substrates. For example, caspase cleavage products of huntingtin and other truncated polyglutamine-containing proteins are known to have increased toxicity in cell culture models (17–19). Thus, preventing the caspase cleavage of huntingtin, atrophin-1 and the androgen receptor protects cells from an apoptotic challenge (20–22). Similarly, caspase cleavage of APP may generate fragments with toxic potential by facilitating the amyloidogenic production of Aβ42 (23).

In this study, we focus on the *in silico* prediction of caspase mediated proteolytic events in human proteins associated with NDs. We used our recently developed, accurate caspase substrate prediction algorithm (24) to understand the importance of the caspase cleavage events and their regulation in NDs. We created CaspNeuroD, a database of predicted caspase cleavage sites in human proteins involved in NDs. This database integrates information about the caspase cleavage positions; their conservation in orthologous proteins in 11 organisms and information about the single nucleotide polymorphisms (SNPs) and PTMs that may modulate caspase mediated proteolytic events.

## Methods

**Figure 1.**
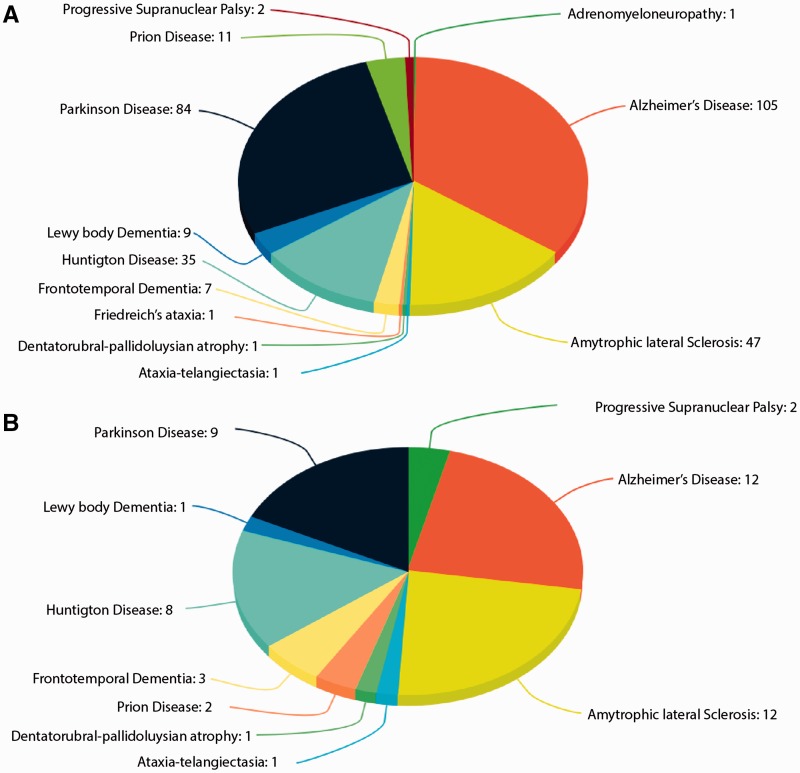
Proteins in ND. **(A)** The number of proteins known to be associated with ND; **(B)** The number of proteins associated with ND known to be caspase substrates.

Human neurodegerative diseases related proteins were extracted from the literature-based resource ‘NeuroDNet’ (25). We collected >300 genes, which were reported in clinical studies of NDs (Figure 1A). We mapped them to verified set of human proteins from the Uniprot database (26) and retrieved 249 protein sequences for further analysis. We applied machine learning prediction method and the Random Forest (RF) classifier, as implemented in our CaspDB (24), to predict caspase cleavage sites in these proteins. The cleavage prediction method provides appropriate cleavage efficiency probability scores in the range 0–1 for every peptide bond in a protein. The score above the 0.5 threshold indicates that the peptide bond is cleaved. The CaspDB RF prediction model was constructed by combining the positional weight matrix characterizing each P5 to P3’ positions and information related to predicted structural features, including secondary structure and disorder parameters. The CaspDB RF model is trained using known human caspase cleavage sequences from the curated CASBAH database (27). The prediction model has been evaluated and discussed in our recent publication (24).

The results of the caspase cleavage predictions in proteins related to NDs are collected and presented in ‘CaspNeuroD’ knowledgebase. It is available to all users at caspdb.sanfordburnham.org/caspneurod.php. The information about proteins sequences in 11 organisms orthologous to human ND proteins were extracted from the OMG browser (28). The curated dbPTM (29) and Humsavar (26, 30) databases were used to obtain information about experimentally known PTMs and SNPs in each protein, respectively. We also located and present the positions of cleavage sites using graphical representation of the protein domains architecture according to information retrieved from the Pfam database (31).

CaspNeuroD is currently configured on an Apache (CentOS) server hosted at the SBP Medical Discovery Institute. It has been developed based on a combination of three layers. The underlying layer is the MySQL database system that stores all the information about the putative cleavage sites in proteins related to NDs and their orthologs along with the Pfam domains, SNPs and PTMs in the back-end. The intermediate layer is an Apache-PHP application that receives the query and connects to the database to fetch data from the upper layer, which comprises populated HTML and PHP pages, to the web browser client. ClustalW (32) and Jalview (33) were implemented to show the pairwise alignment and multiple sequence alignment, respectively.

## Findings

CaspNeuroD is a web-based, platform-independent, database of predicted caspase cleavage sites in 249 human proteins related to NDs. Among them we found 51 proteins, which are already known caspase substrates and are included in two caspase substrates databases: CASBAH and Degrabase (34). This subset of proteins is involved in 10 types of NDs (Figure 1B). The list of known caspase substrates involved in various types of NDs is presented in Supplementary file 1.

Recently, Julien *et al.* (35) published the results of the quantitative MS-based analysis of substrates of Caspase -2 and -6. In this article, it was demonstrated that some 235 and 871 proteins have been detected as substrates of Caspase-2 and -6, respectively. Among them 128 and 553 substrates of Caspase-2 and -6, respectively, have not been previously reported. These new substrates have been added to Degrabase database. Caspase-6 is implicated in ND, including Huntington’s and Alzheimer’s diseases (36). In the data presented by Julien *et al.* there are 24 and 23 substrates in common with our CaspNeuroD for Caspase-2 and -6, respectively. Among them 16 substrates are common for both of these enzymes.

On the front page of the CaspNeuroD database the user can choose one out of 12 NDs listed from a drop down query box in order to retrieve a list of appropriate proteins. All the proteins associated with a selected ND are shown in tabular form. This table contains Uniprot identifier, gene name, gene id (linked to NCBI gene information), chromosome location, onset, and literature reference. To retrieve information about predicted caspase cleavage sites in a protein user can click on the individual Uniprot identifier. All the results related to proteolytic events are shown in a tabular form. The result page contains the information about: (i) the caspase mediated cleavage position (P1) with score values (in the range 0–1) arranged by default in descending order of score value, predicted secondary structure (α-helix: ‘H’, β-sheet: ‘E’, loop: ‘_’) and disorder characteristics (‘.’-ordered or ‘*’-disorder) for each residue at every P5-P3’ position, and substrate prediction class (‘yes‘for cleavages with probabilities scores above 0.5 or ‘no’ otherwise), (ii) the presence of a signal peptide, description of the domains structure in graphical and tabular form according to PFAM annotation, (iii) the list of PTMs and SNPs, including disease annotation of the latter, (iv) multiple sequence alignment with available orthologs.

We used the SignalP v.4.0 program (37) to evaluate the presence of signal peptide characterizing secreted proteins. We also used PFAM knowledgebase for domain annotation to determine the inter- or intra-domain location of cleavage sites. If a given protein is experimentally annotated as caspase substrate and is reported in one of four known databases (MEROPS, CASBAH, Degrabase and TopFIND (27, 34, 38, 39)) then appropriate links to these databases are provided.

To investigate the conservation of a substrate’s cleavage sites in other organisms, orthologous proteins from 11 organisms (Supplementary file 2) were retrieved. A special ‘Compare’ button is available for evaluating pair-wise comparison of cleavage sites between a given substrate and its orthologous proteins. A standard ClustalW pair-wise alignment aids in analysis of the conservation of cleavage sites. To display the multiple sequence alignment of a substrate and all its orthologous proteins, a ‘Start Jalview’ button is provided. The output page includes a list of SNPs and PTMs, with appropriate annotations, because both types of protein modification may influence the outcome of caspase-mediated proteolysis.

In summary, we provide a user-friendly knowledgebase for retrieving information about potential caspase cleavage sites for all verified human proteins associated with NDs. This database provides additional information that would be helpful in generating new hypotheses and in verification of new experimental findings concerning caspase-mediated cleavages of putative substrates. Information about cleavages in orthologous proteins is useful in assessing conservation of the cleavage positions across species, and thus assessing the confidence of the prediction. Overall, our database will complement ongoing experimental efforts in identifying role of new caspase substrates and further our understanding of the biochemistry of caspase-mediated substrate cleavages in NDs.

As more information about caspases and their substrates related to ND becomes available we will update our database.

## Supplementary data

Supplementary data are available at *Database* Online. 

## Funding

This work was supported by the National Institutes of Health (R01GM098835).

*Conflict of interest:* None declared.
